# Dysregulation of the Tau-Microtubule-End-Binding Protein Axis in Alzheimer’s Disease and Related Tauopathies

**DOI:** 10.3390/ijms27125467

**Published:** 2026-06-17

**Authors:** Mahmudul Hasan, Kholoud Abd-ElRaouf, Sophia R. Moran, Chih Hung Lo

**Affiliations:** 1 Department of Biology, Syracuse University, Syracuse, NY 13244, USA; 2 Interdisciplinary Neuroscience Program, Syracuse University, Syracuse, NY 13244, USA

**Keywords:** Alzheimer’s disease, hyperphosphorylation, tau oligomerization, tau aggregation, liquid-liquid phase separation, end-binding proteins, cytoskeleton, microtubule stability, neurodegenerative disorders, therapeutic strategy

## Abstract

Alzheimer’s disease (AD) and related tauopathies are marked by progressive cognitive decline, synaptic dysfunction, and neuronal loss. The microtubule (MT)-associated protein tau, encoded by the *MAPT* gene, plays a central role in neurodegenerative pathology. Although the dissociation of hyperphosphorylated tau from MTs and their subsequent aggregation has been extensively studied, the broader landscape of other MT-associated proteins remains largely underexplored. Among these, the end-binding protein (EBP) family, which comprises MT plus-end-tracking proteins, has emerged as a critical regulator of MT dynamics and stability. EBPs modulate MT polymerization, interact with various MT-associated proteins, and influence cytoskeletal organization. Recent studies suggest that pathological tau impairs end-binding protein 3 (EB3) function by limiting its localization to MT plus-ends and inhibiting EB3-mediated MT elongation and stability. In addition, EB1 appears to interfere with tau aggregation in an in vitro study involving biomolecular condensates. Dysregulation of dynamic tau-MT-EBP interactions may result in structural and functional consequences throughout the entire network, potentially increasing MT instability under neurodegenerative conditions. Hence, the tau-MT-EBP network is an emerging mechanistic axis for advancing the understanding of physiological processes, disease pathology, and therapeutic interventions. In this review, we summarize recent advances in understanding the tau-MT-EBP axis and highlight the molecular mechanisms underlying key pathological interactions within this network. Finally, we discuss current therapeutic strategies and future directions for targeting this dynamic axis to mitigate AD and related tauopathies.

## 1. Introduction

Alzheimer’s disease (AD) and related tauopathies are neurodegenerative diseases (NDDs) characterized by progressive cognitive decline, synaptic dysfunction, and neuronal death [1]. Globally, dementia affects more than 55 million individuals, with AD constituting the most prevalent form and accounting for 60–70% of all dementia cases [2,3,4]. Aggregation of the microtubule (MT)-associated protein tau, encoded by the *MAPT* gene, through oligomerization and fibrillization, represents a pathological hallmark of AD and related tauopathies [5,6]. The human central nervous system (CNS) expresses six tau isoforms, generated by its alternative splicing of exons 2, 3, and 10 [7]. Inclusion or exclusion of exon 10 produces tau variants containing either three (3R) or four (4R) microtubule-binding domains (MTBDs). Under physiological conditions, these isoforms are present at approximately equal levels and stabilize MTs in axons, supporting MT assembly, growth, and stability [8,9], thereby preserving overall neuronal structure and function [10]. Disruption of the normal 3R/4R tau isoform ratio is associated with NDDs and this feature displays significant molecular and clinical heterogeneity [11,12]. For instance, AD is characterized by the accumulation of both 3R and 4R tau isoforms within neurofibrillary tangles [13,14], while frontotemporal dementia (FTD) may exhibit predominantly 3R, 4R, or mixed isoform pathology, depending on the underlying genetic and molecular subtype [15,16]. In addition, corticobasal degeneration and progressive supranuclear palsy are primarily 4R tauopathies [17,18] and Pick’s disease is defined by tangles containing 3R tau isoforms [18]. These distinctions suggest that tau-mediated MT dysfunction and its downstream effects vary across tauopathies, underscoring the importance of elucidating the role of tau across diverse disease contexts.

Besides tau pathology, excessive production and accumulation of beta-amyloid (Aβ) also initiate a series of pathological events that drive AD development [19,20]. A recent study reported that MT provides an intriguing nexus for pathological interactions between tau and Aβ [21]. Importantly, Aβ has also been shown to bind MT with an affinity comparable to tau, potentially competing and displacing tau from MTs, thus promoting tau phosphorylation and aggregation [21]. MT remodeling pathways have also been reported as an early factor that drives morphological change, regulates trafficking, and controls cytokine responses in glial cells under neuroinflammatory conditions [22]. Metabolic alterations, such as mitochondrial impairment [23] and lysosomal dysfunction [24,25], have been implicated in increasing oxidative stress and the accumulation of toxic intrinsically disordered protein aggregates. Mitochondria and lysosomes depend on the MT network and associated motor proteins for intracellular trafficking and proper spatial distribution [26,27,28]. Additionally, dysregulation of lipid metabolism impairs neuronal function, underscoring the importance of the role of MT in lipid homeostasis [29]. These findings suggest the interconnected roles of MT with various cellular components in both normal physiology and disease progression.

The development of the mammalian CNS depends on coordinated neuronal generation, migration, and differentiation, all of which require a dynamic MT cytoskeleton. MTs constitute the structural and functional backbone of neurons and exhibit dynamic instability, which is regulated by MT-associated and plus-end tracking proteins (+TIPs) [30,31,32,33]. Structurally, MTs are hollow cylindrical assemblies composed of parallel protofilaments formed from α- and β-tubulin heterodimers arranged in a head-to-tail orientation, resulting in a pseudo-helical lattice [34]. MTs undergo dynamic instability, alternating between polymerization and depolymerization [35]. During growth, tubulin heterodimers are added to the ends of MTs in their guanosine triphosphate (GTP)-bound form. This is followed by β-tubulin hydrolyzing GTP to guanosine diphosphate (GDP), which forms a less stable MT lattice. The growing plus-end is typically stabilized by a transient GTP cap which protects it from disassembly. Exposure of GDP-bound tubulin promotes rapid depolymerization, a process known as catastrophe. MTs can then switch back to a growth phase, referred to as rescue [36]. Based on its stabilizing function, tau binds preferentially to GDP-tubulin within the MT lattice, rather than to GTP-tubulin at the plus-end cap [36]. Tau is closely associated with the dynamic properties of MTs [24,25], which are critical for neuronal development, synaptic function, and plasticity [37]. Hyperphosphorylated tau dissociates from MTs, leading to toxic aggregates inside neurons and ultimately disrupting axonal transport and impairing synaptic function in AD and related tauopathies [38,39]. Moreover, dysregulation of MT functions, as observed in NDDs such as AD and tauopathies, impairs axonal transport and contributes to progressive neuronal degeneration [40,41].

In addition to aberrant tau aggregation and its pathological associations with MTs, other MT-associated proteins are also implicated in neurodegenerative pathology [42]. MT end-binding proteins (EBPs), which are key +TIPs, have been extensively studied for their localization to the growing plus-ends of MTs and their role in regulating MT dynamics by recruiting co-factor and scaffold proteins [43,44,45]. This EBP family comprises three members, namely, end-binding protein 1-3 (EB1-3). EB1 is ubiquitously expressed and supports persistent MT growth by suppressing catastrophes [30], whereas EB3 is enriched in the brain and increasingly upregulated during neuronal maturation [46]. Both EB1 and EB3 have been reported to contribute to the dysregulation of MT dynamics and cytoskeletal collapse [47]. Loss of tau function has been shown to reduce MT plus-end localization of EB1 in frog neurons and N1E-115 mouse neuroblastoma cells, as well as primary mouse cortical neurons [47] and fibroblast cells [48]. A similar effect is also observed in the tau-EB3 association, where tau reduces EB3 intensity and interferes with EB3 tracking along the MT elongation [48]. Disequilibrium of this interaction, as found in AD and tauopathies, may enhance MT depolymerization, disrupt dendritic spine stability, and suppress synaptic activity [48,49]. Compared to EB1 and EB3, EB2 exhibits a stronger association with the MT lattice, and its expression is critical for MT reorganization during the initial phase of epithelial differentiation [50,51]. While the role of EB2 in neuronal function remains to be investigated, recent studies highlight the essential roles of EB1 and EB3 in both physiological and pathological conditions, particularly in destabilizing MTs and in synaptic dysfunction in AD and tauopathies.

The tau-MT-EBP axis forms a dynamic and interconnected system required for critical neuronal processes, such as neuronal growth, structural integrity, and intracellular transport. Disruption of any single interaction within this axis, such as the loss of tau-tubulin or tau-EB1/3 binding, may cause structural and functional consequences across the entire axis, potentially amplifying MT instability in neurodegeneration. However, the precise mechanisms and dynamic regulation of the tau-MT-EBP axis under physiological conditions are not fully understood, nor is it clear how these interactions are dysregulated by pathological tau or EBPs in NDDs. In this review, we provide a comprehensive overview of the dynamic tau-MT-EBP axis and describe the molecular mechanisms underlying the pathological tau-EBP interplay, including the role of EBPs in modulating the phase behavior and aggregation of different tau species. We further explore how dysregulation of this axis contributes to dendritic spine loss, synaptic dysfunction, impaired neuronal connectivity, and its potential association with neuroinflammation. Finally, we present potential therapeutic strategies and future perspectives on targeting the tau-MT-EBP axis in AD and tauopathies to preserve cytoskeletal integrity and mitigate neurodegenerative pathology.

## 2. Molecular Mechanisms Underlying the Tau-MT-EBP Axis

In the tau-MT-EBP axis, tau and EBPs show dynamic changes in both expression and localization, which regulate MT dynamics and neuronal function. Tau and EB3 levels increase during development, whereas EB1 levels remain relatively constant [49,52]. Although the mechanism by which EB1 influences pathological tau aggregation is unclear, its presence at MT growth tips and evidence from tau pathology studies highlight its importance in AD and tauopathies. In undifferentiated neuroblastoma cells with moderate tau expression, tau and EBPs do not show significant colocalization. Upon differentiation and neurite extension, both proteins become enriched and partially colocalize [47]. Tau is necessary for proper EB localization within axons, as its depletion disrupts EB1 accumulation at MT bundles in the medial to distal axon [47]. Both EB1 and EB3 are implicated in neurite extension, and changes in tau expression or localization are mirrored by alterations in EB1 and EB3 distribution [53,54]. These observations suggest that tau-mediated recruitment of EBPs regulates MT stability during axon outgrowth. Consistently, delayed axon formation occurs in tau-deficient neurons [55,56].

Structurally, EBPs are organized into an amino-terminal calponin homology (CH) domain, an intrinsically disordered region (IDR), a coiled-coil domain, a four-helix bundle, and a disordered tail ending with a carboxy-terminal EEY/F motif. The four-helix bundle and part of the tail form the EBP homology domain [57]. The C-terminal region contains binding sites for various +TIP partners. While the CH domain and IDR are sufficient for recognizing and tracking growing MT ends, interaction between the CH-IDR domains and the charged C-terminal region is required to fine-tune EBP specificity for MT tips [58]. EBP-interacting molecules fall into two main groups: cytoskeletal-associated protein glycine-rich (CAP-Gly) proteins and SxIP-containing proteins. CAP-Gly domains feature a distinct hydrophobic region with a highly conserved GKNDG motif and several glycine residues [31,59]. SxIP-containing proteins have one or more Ser/Thr-X-Ile-Pro motifs within an unstructured region rich in serine, proline, and basic residues [60,61]. These proteins regulate various aspects of MT dynamics and function [62,63].

Early hypotheses suggested that MT-EB1 interactions are regulated by the positively charged EB1 N-terminal MT-binding domain and the negatively charged C-terminal acidic tail of tubulin [64]. Removing the C-terminal tyrosine does not affect EB1 binding [65], which initially led researchers to unclear evidence on the involvement of the tubulin tail in MT-EB1 interactions. However, later studies showed that MT-EB1 interactions can be disrupted by salt, supporting electrostatic interaction. Furthermore, removal of the tubulin acidic tail significantly reduces EB1 binding to MTs, confirming that the tubulin tail mediates part of the interaction [64]. Both tau and EB1 bind, at least in part, to MTs via the tubulin C-terminal tails [66,67,68]. Additionally, some studies find that tau and EB1 can interact directly [47], with the auto-inhibitory tail of EB1 mimicking the tubulin C-terminal tail [64,69]. This suggests that tau may bind to this tail and activate EB1, resulting in synergistic binding of both proteins to MTs [70].

Studies suggest that tau repeat motifs are essential for the tau-EB1 interaction. For example, the removal of conserved basic repeats (R1–R2) from the C-terminal region of tau completely abolishes the tau-EB1 interaction. In contrast, the acidic N-terminal domain does not affect tau-EB1 binding [48]. Although tau 3R and 4R isoforms exhibit distinct MT-binding properties and their imbalance characterizes several tauopathies [71], direct tau isoform-specific effects on EBP interactions have not yet been clearly established. The MT-stabilizing function of tau is also regulated by phosphorylation [72]. Since EB1 interacts with tau MTBDs, post-translational modifications in this region are likely to affect the extent and specificity of the tau-EB1 interaction. Phosphorylation at serine 262, within the first repeat in the MTBD, occurs under both physiological and pathological conditions [73]. A phosphorylated tau mutant, where serine 262 is replaced by glutamate (tauS262E), shows about a two-fold reduction in interaction with EB1. This highlights the sensitivity of the tau-EB1 interaction to site-specific phosphorylation and suggests a mechanism for tau dysfunction in disease pathology. Studies also showed that tau interferes with EB1 localization, affecting its MT-binding and assembly-promoting properties [48]. In addition to phosphorylation at Ser262, modifications at other sites, such as Thr231 and Ser235, have been identified as critical for MT-binding function [74], although their specific roles in EBP interactions are unknown and remain to be investigated. These findings highlight the importance of investigating tau-EB1 interactions in neurodegeneration, including AD.

**Figure 1:**
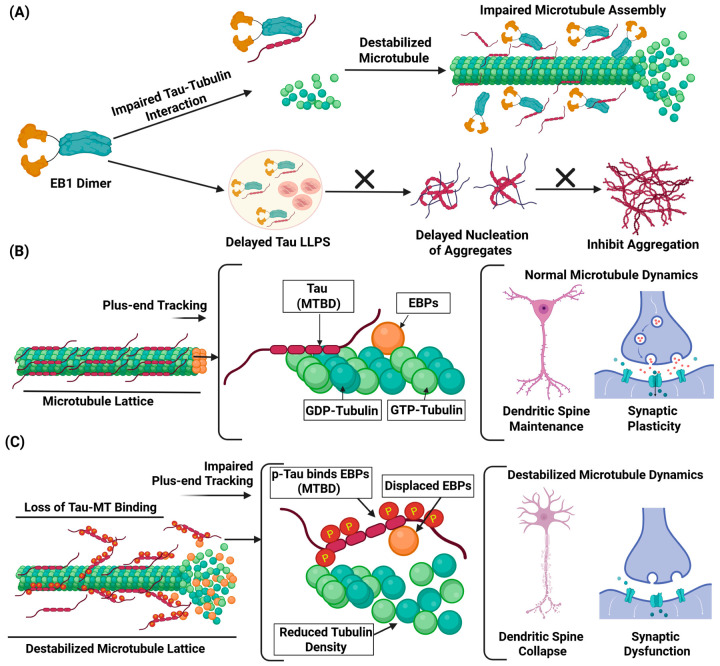
Dynamic interplay underlying the Tau-MT-EBP axis. (**A**) EB1 plays dual roles, promoting MT growth and modulating tau-MT interactions. EB1 interacts with tau to restrict its transition into oligomeric and fibrillar assemblies; however, this interaction may compromise MT stabilization. (**B**) EBPs facilitate the physiological organization of tau and MT. In healthy neurons, EBPs are associated with tau at the MT lattice to maintain MT dynamics, neuronal morphology, and synaptic stability. (**C**) Tau-dependent displacement of EBPs leads to destabilized MT dynamics and related neuronal dysfunction. Created in https://BioRender.com.

EB1 modulates tau aggregation kinetics, as shown by reduced thioflavin-S fluorescence during heparin-induced tau aggregation in the presence of EB1. Specifically, EB1 inhibits tau aggregation by reducing oligomer and higher-order aggregate formation, with a half-maximal inhibitory concentration of 4.3 ± 0.2 µM [75]. The study also examined tau liquid–liquid phase separation (LLPS) with varying EB1 concentrations. LLPS typically initiates tau aggregation and facilitates nucleation [75,76,77]. EB1 increases tau dynamics in phase-separated droplets, suggesting a delay in tau aggregation [75]. These results demonstrate that EB1 modulates tau-MT interactions and regulates tau aggregation and phase behavior (Figure 1A). However, the proposed role of EB1 in modulating tau aggregation is currently supported by in vitro evidence. Further neuronal and in vivo studies are necessary to validate this mechanism and establish its significance in both physiological and pathological contexts.

Importantly, EB1 has been shown to regulate absent in melanoma 2 (AIM2) inflammasome–induced cytokine secretion through autophagy-mediated shedding following speck formation, a process that may be regulated by AMP-activated protein kinase [78]. Although this study illustrates the regulation of AIM2 inflammasome activity by EB1 in non-neuronal systems, AIM2 activation has been implicated in synaptic dysfunction and cognitive impairment in various neurodegenerative disease models. For example, overexpression of AIM2 inflammasome leads to synaptic and cognitive impairments in C57BL/6 mice, mirroring those seen in AD models [79]. In addition, conditional knockout of microglial AIM2 restores cognitive and synaptic function in AD mice [79]. Similarly, in a mouse model of vascular dementia, the AIM2 inflammasome mediates key neuropathological changes and cognitive deficits [80]. However, there is currently no direct evidence connecting EB1-mediated regulation of the AIM2 inflammasome to neuroinflammation in AD and tauopathies. Therefore, further research is required to elucidate the effective link between EB1-regulated AIM2 inflammasome activity and neurodegenerative processes in relevant mouse models.

The functions of EB2, in contrast to EB1 and EB3, have been less extensively investigated in the context of neurodegeneration or tauopathies. In humans, EB2 has been implicated in circumferential skin creases Kunze type, a rare disorder characterized by facial dysmorphism, limb skin creases, microcephaly, and intellectual disability [81,82]. However, some studies report that EB2 is relatively highly expressed in the vertebrate brain [83] and is involved in mitotic defects [83] and p53-induced apoptosis [84], suggesting its potential role for future investigations. Given that microtubule dysfunction and aberrant apoptotic signaling are established pathological features of many NDDs, alterations in EB2-mediated pathways may contribute to neuronal vulnerability and disease progression. Although direct evidence linking EB2 to specific NDDs remains limited, its prominent expression in neural tissues and involvement in cytoskeletal regulation and apoptosis suggest a potentially underexplored role in maintaining neuronal homeostasis. These observations underpin the need for further investigation of EB2 functions in microtubule instability, neuroinflammation, and neurodegeneration.

EB3 is highly abundant in neurons and supports dendritic spine growth and morphology [47,48,85]. It is also widely used to visualize dynamic MTs in neurons [86]. Changes in EB3 expression significantly affect dendritic spine morphology. Overexpression of EB3 increases the proportion of mushroom-shaped spines, which are associated with strong synaptic connections and memory storage. Elevated EB3 levels can restore spine deficits in hippocampal neurons from the PS1-M146V-KI AD mouse model [87]. However, these mice do not express human amyloid precursor protein or produce human Aβ and thus do not develop amyloid-related toxicity. Addressing this, a subsequent study evaluated the neuroprotective potential of EB3 under low amyloid toxicity, focusing on its effects on dendritic architecture through morphometric analyses. The results show that maintaining appropriate expression is critical for normal dendritic branching and mature dendritic spines [86]. These outcomes highlight the need for further investigation of EB3 function in more physiologically relevant disease models. Studies also found that pathological tau restricts EB3 binding to MT plus-ends by directly associating with EB3, thereby suppressing EB3-mediated MT elongation and stability. Specifically, the C-terminal region of EB3 interacts with tau and serves as a critical region for the inhibitory effect of tau on EB3 tracking at the MT end [47,48]. Tau-EB3 dynamics are implicated not only in AD pathology but also in other tauopathies, such as FTD. Most tau mutations associated with FTD are localized within the MTBD [39,88]. A recent study has shown that the FTD-linked tauV337M disrupts activity-dependent cytoskeletal plasticity at the axon initial segment (AIS) [89]. Mechanistically, tauV337M mutation increased binding affinity for EB3 compared to wild type tau, resulting in greater accumulation of EB3 within the AIS. This abnormal enrichment impairs AIS structural plasticity. Nuclear magnetic resonance analyses confirm that tau interacts with EB3 through its MTBD [89]. Elevated EB3 levels cause AIS shortening and inhibit AIS plasticity, while reducing EB3 expression restores both AIS plasticity and neuronal activity homeostasis in neurons expressing tau V337M [89].

Interestingly, a peptide fragment of activity-dependent neuroprotective protein, referred to as NAP, enhances tau-MT interactions and promotes EB1 and EB3 binding with tau [90]. For example, research demonstrated that NAP increased endogenous EB1 comet density in the N1E-115 neuroblastoma neuronal model and facilitated EB3 homodimer formation, while reducing EB1-EB3 heterodimer levels. Additionally, NAP promoted tau-EB1/3 interactions, resulting in the recruitment of EB1/EB3 and tau to MT under zinc intoxication [90]. Peptides containing SIP and SKIP motifs have also demonstrated interactions with EB1 and EB3 proteins [91]. The study also identified that SKIP and its acetylated derivative, CH_3_CO-SKIP-NH_2_ (Ac-SKIP), influence the crosstalk among tau, EB1, and MT. Both SKIP and Ac-SKIP were observed to enhance MT dynamics, reduce tau-MT dissociation, and increase the interaction between tau and EB1, which correlates with an enhanced association of tau with tubulin [49,92]. Collectively, all these studies highlight the regulatory role of EB3 in maintaining dendritic integrity and synaptic stability (Figure 1B,C).

EBPs are key regulators of MT dynamics and play important roles in neuroprotection. EB1 inhibits tau aggregation and enhances tau dynamics in phase-separated droplets, delaying aggregate formation. Pathological tau sequesters EB3 in the AIS, causing neuronal hyperexcitability and dysfunction. The interplay among tau, EBPs, post-translational modifications, such as phosphorylation, and protective peptides, such as NAP and SKIP, highlights the complex regulation of MT integrity. A deeper understanding of these associations may support the development of therapies targeting the tau-MT-EBP axis in AD and tauopathies. The comparative analysis of tau-EB1 and tau-EB3 interaction studies is summarized (Table 1).

## 3. Targeting the Tau-MT-EBP Axis for Therapeutic Interventions in AD and Tauopathies

Recent studies underscore the significance of tau-MT-EBP dynamics in neuronal function and cognitive processes. The dynamic nature of these components has emerged as a critical area of research for elucidating disease mechanisms and informing the development of targeted interventions. Recent findings related to the tau-MT-EBP axis have identified potential therapeutic strategies to address AD pathology, including other NDDs. This section provides a comprehensive review of current and prospective therapeutic targets, with an emphasis on the tau-MT-EBP axis. The analysis includes strategies to disrupt pathological tau-tau, tau-MT, and tau-EB1/3 interactions, modulate their expressions, stabilize MTs, and enhance EBP activity, all of which may restore cytoskeletal integrity, MT dynamics, and neuronal function to mitigate neurodegeneration.

Targeting tau expression is a promising strategy in the tau-MT-EBP axis, particularly for AD. Antisense oligonucleotides (ASOs) and RNA interference (RNAi) selectively lower pathogenic tau, stabilize MTs, and may reduce cognitive decline [93]. ASOs target human *MAPT* mRNA, while RNAi uses small RNAs to silence specific genes. ASOs show early signs of functional improvement in mild AD [93,94,95]. A clinical study of the tau-targeting ASO BIIB080 (MAPT-Rx) demonstrated substantial reductions in total tau and phosphorylated tau in cerebrospinal fluid, providing clear evidence of target engagement. However, it remains unclear whether tau reduction leads to meaningful clinical benefit, necessitating further evaluation of the effects of MAPT-Rx on the clinical progression of patients with AD and tauopathies [96,97]. On the other hand, RNAi often delivered via adeno-associated viral vectors lowers pathogenic tau isoforms while preserving physiological tau needed for MT stabilization [98].

Maintaining physiological tau is crucial for proper EBP localization and recruitment to MT. Selectively reducing pathogenic tau or maintaining the correct ratio of tau species may restore EB1/EB3 distribution and MT plus-end dynamics [47]. Targeting tau phosphorylation is therefore a promising disease-modifying approach. Sodium selenate (VEL015) reduces hyperphosphorylated tau by activating protein phosphatase 2A (PP2A), which dephosphorylates tau [99]. Preclinical studies show sodium selenate enhances PP2A activity, significantly reducing hyperphosphorylated tau in models of AD, traumatic brain injury, and epilepsy [100,101]. Phosphorylation in the MTBD weakens tau-EB1 associations and disrupts their interaction [48,73]. Inhibiting kinases, such as glycogen synthase kinase-3 beta (GSK-3β), cyclin-dependent kinase 5 (Cdk5), and MT affinity-regulating kinase 2 offers an effective strategy to restore tau binding to MT and promote EB3-mediated MT dynamics [102,103]. Cdk5 and MT affinity-regulating kinase 4 also act together to increase tau phosphorylation and accumulation, promoting neurodegeneration [104]. Lithium, a GSK-3β inhibitor, reduces tau phosphorylation and normalizes axonal transport and MT stability in animal models [105,106]. Although systemic side effects limit their clinical use, lithium still demonstrates neuroprotective effects on MT and cognitive function in AD models [106,107]. Since pathological tau suppresses EB3 plus-end tracking and alters EB3 localization [48], kinase inhibition may also restore EB3-dependent MT elongation and dendritic stability by re-establishing physiological tau-EB3 interactions.

Limiting tau aggregation may also help prevent the pathological sequestration of EBPs into tau aggregates, thereby preserving their regulatory function at microtubule plus-ends. Several tau aggregation inhibitors have shown promise in AD models and other NDDs [108,109,110]. Curcumin reduces tauopathy in animal models, inhibits tau aggregation in vitro, and has been associated with improved memory and attention in treated recipients [111,112,113]. However, despite encouraging preclinical results, its clinical translation remains challenging [114,115]. More recently, curcumin-loaded nanoparticles have demonstrated improved cognitive outcomes in AD models compared with free curcumin [116]. Similarly, LMTX (TRx0237), a methylene blue derivative, crosses the blood-brain barrier (BBB), reduces tau aggregation, and improves cognition in animal models [117,118]. However, multiple Phase III trials in patients with mild-to-moderate AD failed to show clear clinical benefit [119,120]. Although these agents have primarily been studied as tau aggregation inhibitors, their effects within the broader tau-MT-EBP axis remain incompletely defined. Further investigation of how these compounds influence tau-MT interactions and EBP function may therefore yield more mechanistically targeted therapeutic strategies for AD and related tauopathies.

In addition, advanced technologies are used to study the tau-tau interaction and may help target other interactions within the tau-MT-EBP axis. For example, fluorescence resonance energy transfer (FRET) biosensors are engineered to monitor tau oligomers and aggregates and are used to identify therapeutic compounds through high-throughput screening [121]. By using this FRET technology, MK-886 was identified as a compound that perturbs toxic tau oligomer conformation. MK-886 induces conformational changes in tau monomers at the proline-rich region and MTBD and rescues tau-induced cytotoxicity in neuronal cells [122]. FRET biosensors provide real-time, quantitative detection of tau conformational changes, oligomerization, and aggregation dynamics in living cells, enabling precise identification of early-stage toxic tau species. Hence, this technology can also be adapted to study tau-EB interactions or monitor tau and EBP localization relative to MT plus-end markers.

Within the tau-MT-EBP axis, MT provides the main structural platform for tau and EBP association, coordinating distinct functions in health and disease. Several MT-stabilizing drugs (MSDs) have demonstrated potential efficacy in AD pathology [123]. For example, epothilone D [123] and paclitaxel [124] target MTs and prevent their depolymerization. These drugs help preserve axonal transport and dendritic integrity in the presence of tau pathology [123,124]. In tau transgenic mouse models, epothilone D increases MT density, restores axonal transport, preserves synaptic structure, and improves cognition. These therapies address early tau pathology by stabilizing MT. However, a phase 2 trial of Epothilone D was discontinued because preclinical benefits did not translate into sufficient clinical efficacy in humans [125]. In contrast, subsequent studies developed Epothilone analogs that demonstrated efficacy in cancer patients [126,127] and could potentially be applied to neurological diseases. In the case of paclitaxel, treatment initially improved fast axonal transport and neuronal survival, although it was later shown to be unsuitable for neurotherapeutic applications due to peripheral toxicity and limited BBB penetration [128,129]. TPI-287, a second-generation MSD is being developed to improve BBB permeability and reduce toxicity [130]. A Phase 1 trial evaluating TPI-287 in combination with bevacizumab for recurrent glioblastoma demonstrated encouraging results, reporting a median overall survival (OS) of 13.4 months and a 12-month OS rate of 64% [131]. A Phase 2 study of TPI-287 (200 mg/m^2^ every 3 weeks) in combination with bevacizumab (10 mg/kg every 2 weeks) is under development [132].

Peptide-based multi-functional MSDs have also been reported. For example, NAPVSIPQ is a neuroprotective MSD that reduces Aβ and tau burden, improving cognition in animal models [133]. NAPVSIPQ also enhances tau-MT interactions and facilitates EB1/EB3 association with tau [90], suggesting these agents may restore coordinated tau-MT-EBP dynamics. Other peptide-based modulators, SKIP and its acetylated derivative, Ac-SKIP, influence the crosstalk among tau, EB1, and MT. Both SKIP and Ac-SKIP enhance MT dynamics, reduce tau-MT dissociation, and increase tau-EB1 interaction, which correlates with greater tau-tubulin association [49,92]. These modulators may further restore tau-tubulin association and promote MT stabilization. Since EB1 reduces tau oligomerization and delays aggregation [75], increasing tau-EB1 interactions could stabilize MT dynamics and reduce tau toxicity. Similarly, EB3-targeted interventions may restore dendritic and synaptic integrity [86,87]. Because pathological tau sequesters EB3 and impairs its plus-end tracking [49,75], modulating EB3 function, rather than simply increasing its expression, requires further consideration.

Restoring EB1 and EB3 activity is a promising therapeutic strategy in the tau-MT-EBP axis. Studies show that EB3-associated MT dynamics are essential for dendritic spine maintenance and synaptic plasticity, while disruption of EB3-dependent MT dynamics leads to spine destabilization and synaptic loss [49,134,135]. Targeting EBPs through peptide-based approaches has attracted interest as a potential strategy to counteract tau-associated MT dysfunction [136,137]. For example, the SIP motif in the peptides NAPVSIPQ and SALLRSIPA interacts with EB1 and EB3, promoting MT assembly, neuroprotection, and synaptic plasticity [91]. Preclinical models demonstrate that EB3 overexpression normalizes dendritic spine density, enhances MT polymerization, and rescues neurons from tau neurotoxicity [49,85]. Restoring EB3 levels may therefore mitigate tau-induced cytoskeletal disruption at both structural and functional levels. Both pharmacological and genetic approaches show potential to increase EB3 function, reverse tau-promoted MT disassembly, preserve synaptic connectivity, and promote neuronal resilience in early AD pathology. A peptide-based EB3 inhibitor, EBIN, was recently developed through analysis of the EB3 and inositol 1,4,5-trisphosphate receptor 3 interaction at the endoplasmic reticulum membrane and computational modeling. EBIN acts as an allosteric inhibitor of EB3 and has been shown to attenuate environmental stress-induced effects in endothelial cells by restricting pathological calcium signaling [138]. While this approach does not directly target the tau-MT-EBP network, allosteric modulation of EB3 may be relevant for optimizing tau-EBP interactions, which are sensitive to structural and post-translational modifications in both proteins.

**Figure 2:**
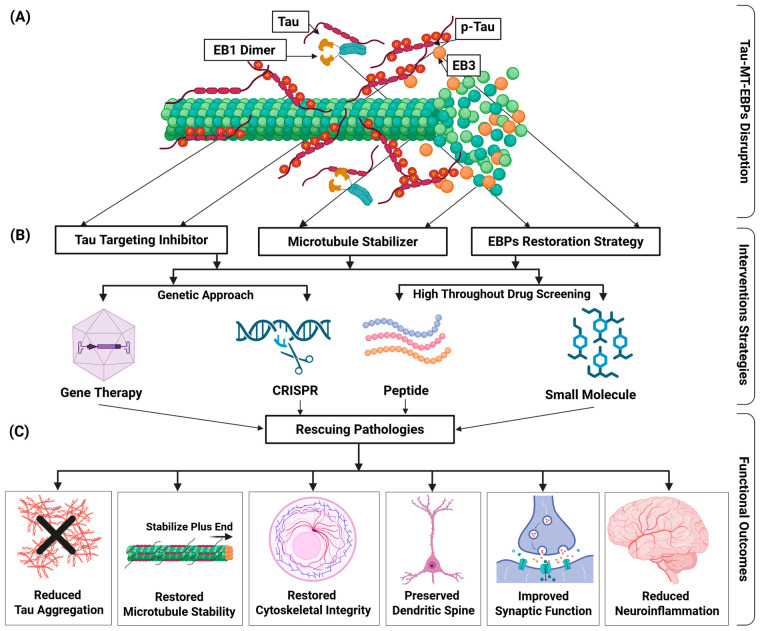
Schematic representation of therapeutic strategies to restore tau-MT-EBP homeostasis. (**A**) In the pathological state, hyperphosphorylated tau accumulates along the MT lattice and the plus-end region, disrupting the normal localization and function of EB1 and EB3, leading to cytoskeletal destabilization. (**B**) Different classes of tau-MT-EBP-targeting therapeutic strategies. (**C**) Rescuing pathologies are expected to ameliorate pathological features, such as reduced tau-tau aggregation, restored MT stability and cytoskeletal integrity, preserved dendritic spine, improved synaptic function, and reduced neuroinflammation. Created in https://BioRender.com.

The strategies outlined above form a comprehensive therapeutic framework in the tau-MT-EBP axis for effective intervention in AD and related tauopathies. Pathological interactions within the tau-MT-EBP network (Figure 2A) can be modulated by therapeutic approaches including tau-targeting inhibitors, MT stabilizers, and EBP restoration strategy (Figure 2B) to rescue neurodegenerative pathologies (Figure 2C). Reducing pathological tau oligomerization and aggregation and inhibiting abnormal tau modifications preserve MT integrity and protects EBP functions. Stabilizing MT improves axonal transport and synaptic function, while activating EBPs supports MT dynamics and dendritic stability. These interconnected strategies highlight the dynamic nature of the tau-MT-EBP axis where its modulation may offer additional protection beyond primary therapeutic targets. This framework supports the development of next-generation therapies for complex NDDs, including AD and tauopathies.

## 4. Summary and Future Perspectives

This review provides detailed insights into the tau-MT-EBP axis, focusing on the mechanistic interactions among its components and potential therapeutic strategies to modulate this crosstalk under pathological conditions. While tau and MT have been extensively studied, recent research highlights the importance of their interaction with EBPs in understanding AD and tauopathies. EB1 and EB3 are relatively well characterized, but EB2 requires further investigation. Notably, recent studies have identified associations between EB2 and apoptosis, raising questions about their roles in neuroinflammation and cell death. Although the tau-MT-EBP axis offers a promising therapeutic framework, several key questions remain. A key unresolved issue is whether EBP dysregulation is a primary driver of tau-mediated neurodegeneration or a secondary consequence of tau pathology. Existing evidence indicates that tau regulates EB1 and EB3 localization and function, and that pathological tau disrupts MT dynamics. Although EB3 is implicated in MT growth, dendritic spine shape, and synaptic stability, its dysfunction may contribute to early cytoskeletal vulnerability or may act downstream of tau-induced MT disruption, both of which could potentially amplify neuronal damage through a feedforward mechanism. However, there is insufficient evidence demonstrating that dysregulation of EBPs precedes the onset of tau pathology. Further research employing temporal, cell-type-specific, and rescue-based approaches to examine the tau-MT-EBP axis is required to clarify the causal relationship between EBP protein dysregulation and tau pathology.

In addition, understanding the structural and biochemical mechanisms underlying tau-EB1 and tau-EB3 interactions, especially across different tau phosphorylation states, is essential [139]. Phosphorylation within the MTBDs alters tau conformation and binding affinity, but the specific phosphorylation sites that regulate tau-EB1 or tau-EB3 associations have yet to be identified [48,73]. Characterizing these modifications and their effects on tau-EBP interactions will clarify how pathological tau disrupts MT dynamics and EBP-dependent plus-end tracking. The increased vulnerability of hippocampal and cortical neurons to tau-EB3 pathology also requires further study. The EB3-associated pathway, which links actin filaments to MT dynamics at dendritic spines and is disrupted early in AD, needs additional investigation as a point of cytoskeletal and synaptic regulation crosstalk [140]. The potential connection between EBPs and neuroinflammation should be prioritized. EB1 regulates AIM2 inflammasome activation, suggesting that tau-EBP interactions may influence immune responses in the diseased brain beyond their role in cytoskeletal regulation [78,141]. Advances in in vivo imaging are crucial for monitoring tau-MT-EBP dynamics in patients. Developing positron emission tomography ligands that distinguish tau isoforms or report on MT stability and EBP localization would improve the ability to track cytoskeletal pathology and assess drug responses over time [142].

Moreover, identifying EBPs as potential therapeutic targets suggests that gene-regulatory strategies could be utilized to restore their expression and function in vulnerable neurons. While CRISPR-based transcriptional activation has been used to upregulate endogenous genes in neuronal systems [143], this technology has not yet been reported for EBPs. Given the critical role of EB3 in microtubule dynamics, neurite outgrowth, and synaptic plasticity, CRISPR-mediated activation of endogenous EB3 constitutes a promising direction for future research. Enhancing EB3 expressions in susceptible neuronal populations could preserve MT dynamics and synaptic integrity in neurodegenerative conditions; however, this hypothesis requires experimental validation. It is critical to determine whether different therapeutic strategies can selectively target specific interactions without affecting normal physiological functions. Some therapeutic strategies targeting pathological modifications of tau, MT, or EBPs have been reported, but a more comprehensive understanding of the coordinated mechanisms within the tau-MT-EBP network is still needed for effective interventions. Future research should focus on advanced methodologies to identify compounds that specifically target components of the tau-MT-EBP axis. These compounds should be validated for efficacy in complex biological systems, including cellular and animal models. It is also important to assess whether combinatorial therapeutic strategies can synergistically restore cytoskeletal integrity and synaptic function in complex environments of AD and tauopathies.

## Figures and Tables

**Table 1:** Comparative analysis of tau-EB1 and tau-EB3 interactions in physiological and pathological conditions.

Feature	Tau-EB1	Tau-EB3	References
Expression	Ubiquitously expressed	Enriched in the brain	[46,48,52]
Molecular interaction	Binds to tau MTBDs	Binds to tau MTBDs	[46,48]
Regulation by phosphorylation	Tau phosphorylation at Ser262 reduces Tau-EB1 interaction	Role of phosphorylation in altering interaction has not been reported	[48,52]
Physiological function	MT stability during axon outgrowth and neurite extension	Dendritic spine growth and morphology, MT dynamics, synaptic plasticity, and neuronal connectivity	[46,49,54,85,87]
Effect of pathological tau	Pathological tau interferes with EB1 localization	Pathological tau restricts EB3 binding to MT plus-ends	[48,89]
Effects on tau aggregation	In vitro study finds EB1 inhibits tau aggregation	No direct role has been reported	[75]
Characterization methods	Direct binding; phosphorylation dependent interaction; localization analyses; tau aggregation and LLPS assays	Localization analyses; MT plus-end tracking assays; dendritic spine analyses; AIS plasticity studies	[46,48,75,85,87,88,89]
Experimental models	Frog neurons; N1E-115 neuroblastoma cells; primary mouse cortical neurons; fibroblasts; in vitro aggregation/LLPS systems	Neuronal cells; hippocampal neurons; PS1-M146V-KI neurons; tauV337M-expressing neurons	[46,48,75,85,87,88,89]

## Data Availability

No new data were created or analyzed in this study. Data sharing is not applicable to this article.
